# *Moringa oleifera* and Blood Pressure: Evidence and Potential Mechanisms

**DOI:** 10.3390/nu17071258

**Published:** 2025-04-03

**Authors:** Francesca Menichetti, Chiara Berteotti, Vittoria Schirinzi, Carolina Poli, Roberta Arrighi, Alessandro Leone

**Affiliations:** 1 International Center for the Assessment of Nutritional Status and the Development of Dietary Intervention Strategies (ICANS-DIS), Department of Food, Environmental and Nutritional Sciences (DeFENS), University of Milan, 20133 Milan, Italy; 2 PRISM Lab, Department of Biomedical and Neuromotor Sciences, Alma Mater Studiorum University of Bologna, 40126 Bologna, Italy; 3 Endocrinology and Care of Diabetes Unit-Azienda Ospedaliero-Universitaria S. Orsola Malpighi, Alma Mater Studiorum University of Bologna, 40126 Bologna, Italy; 4 Professional Development and Implementation of Research in Health Professions Unit, IRCCS Azienda Ospedaliero, Universitaria di Bologna, 40126 Bologna, Italy; 5 IRCCS Istituto Auxologico Italiano, Clinical Nutrition Unit, Department of Endocrine and Metabolic Medicine, 20100 Milan, Italy

**Keywords:** *Moringa oleifera*, hypertension, blood pressure, traditional medicine, developing countries

## Abstract

The prevalence of hypertension is increasing worldwide, in particular in developing countries. Anti-hypertensive drugs are commonly used to treat hypertension. However, in developing countries, where access to health care is scarce and the supply system is poor, anti-hypertensive drugs may not always be available. *Moringa oleifera* is a plant widely found in developing countries, with its leaves, seeds, flowers, roots, and pods used both for nutritional purposes and in traditional medicine to treat various diseases, including hypertension. This review summarizes the evidence, both in animal and human models, about the antihypertensive effects of different parts of *M. oleifera*, discusses possible mechanisms of action, explores its bioactive compounds with potential antihypertensive properties, and highlights the limitations of its use as a hypotensive agent. Many preclinical studies attribute antihypertensive properties to *M. oleifera*, particularly the leaves. However, it is premature to draw firm conclusions, as there is a great lack of randomized controlled trials demonstrating its real efficacy. The mechanisms of action and the compounds responsible for the hypotensive effect have not yet been fully elucidated. Therefore, further clinical trials showing its efficacy are strongly required before promoting Moringa for therapeutic purposes. At present, Moringa remains a plant with nutritional and pharmacological potential.

## 1. Introduction

Hypertension, defined as a systolic blood pressure of 140 mm Hg or higher or a diastolic blood pressure of 90 mm Hg or higher [1], is a leading preventable risk factor for cardiovascular disease (CVD) and all-cause mortality worldwide [2]. Between 1990 and 2019, the global prevalence of hypertension nearly doubled, reaching 1.13 billion individuals, with the highest burden observed in low- and middle-income countries [3].

Lifestyle modifications, including salt restriction, the moderation of alcohol consumption, the consumption of vegetables, fruits, and low-fat dairy products, weight reduction, regular physical exercise, and smoking cessation, have proven effective in reducing blood pressure. However, implementing these changes can be challenging and not implemented [4], or not sufficient to bring blood pressure values back to normal limits. Consequently, the use of oral medications is recommended [1]. These drugs are effective but can have side effects and may not always be available, especially in developing countries with fragile healthcare systems and inadequate supply chains [5].

The food environment offers another promising avenue for the prevention and treatment of hypertension. Medicinal plants, used in traditional medicine for centuries, contain bioactive compounds that can help regulate blood pressure. These plants are affordable, accessible, and have minimal side effects, making them an attractive option, especially in developing countries. Among them, *Moringa oleifera*, a plant widely cultivated in tropical and subtropical regions, has gained attention for its nutritional [6] and medicinal properties [7,8]. In particular, its leaves are abundant in proteins, minerals (potassium, calcium, magnesium, iron), vitamins (β-carotene, α-tocopherol), dietary fiber [9,10,11], and bioactive compounds [6,12,13]. Due to this rich composition, they are widely utilized in traditional medicine across developing countries to treat various ailments, including diabetes, cardiovascular diseases, and hypertension [14,15,16].

While several reviews have examined the health benefits of *M. oleifera*, including its effects on cardiac function [17] and cardiometabolic disorders [18], to our knowledge, no recent review has specifically focused on its hypotensive effects by analyzing both preclinical and clinical studies. This review provides a comprehensive overview of the evidence regarding *Moringa*’s impact on blood pressure, explores the potential physiological mechanisms and bioactive compounds responsible for its hypotensive effects, and discusses the limitations of the current research. We also address challenges in using *M. oleifera* as an antihypertensive therapy and propose directions for future studies.

## 2. Physiological Regulation of Blood Pressure

Arterial blood pressure is regularly measured in patients to assess cardiovascular health. It is expressed in millimeters of mercury and consists of systolic and diastolic measurements. Systolic pressure represents the maximum pressure in the arteries when the heart contracts to pump blood, while diastolic pressure is the minimum pressure when the heart relaxes between beats [19]. Arterial pressure is closely related to cardiac output, arterial elasticity, and peripheral vascular resistance. It can be easily influenced by various activities, and ideally, blood pressure should range from 90/60 mmHg to 120/80 mmHg. Maintaining this range is crucial for health. Stage 1 hypertension is classified between 140/80 mmHg and 159/99 mmHg [20], while stage 2 hypertension falls within the range of 160/100 mmHg to 179/109 mmHg [21]. Hypertensive urgency occurs with readings over 180/120 mmHg, and hypertensive emergency involves life-threatening symptoms and organ damage [22]. Hypotension is defined as a blood pressure lower than 90/60 mmHg [23]. The body uses several mechanisms to regulate blood pressure, including the baroreceptor reflex, antidiuretic hormone, and the renin–angiotensin–aldosterone system (RAAS).

### 2.1. Baroreceptor Reflex

Baroreceptors, located in blood vessels, play a key role in regulating blood pressure by responding to vessel wall stretch. These mechanoreceptors send sensory information to the central nervous system, influencing vascular resistance and cardiac output. There are high-pressure and low-pressure baroreceptors. High-pressure baroreceptors are found in the carotid sinus and aortic arch. They respond to blood pressure changes, sending signals via the glossopharyngeal and vagus nerves, respectively.

Activation of these receptors leads to a response that influences sympathetic activity: increased blood pressure suppresses sympathetic activity, while decreased blood pressure (e.g., in hypovolemic shock) reduces baroreceptor firing, raising sympathetic activity to increase blood pressure [24]. Low-pressure baroreceptors are located in large veins, pulmonary vessels, and the right atrium and ventricle, responding mainly to volume changes. Decreased firing from these receptors triggers the release of antidiuretic hormone, renin, and aldosterone to regulate blood pressure [25]. Although baroreceptors control short-term blood pressure fluctuations, their role in long-term regulation is limited, as they reset to a new pressure level over time. However, experimental studies suggest they can still influence long-term blood pressure, especially by modulating kidney sympathetic nerve activity [26]. In hypertension, baroreceptor sensitivity decreases due to carotid sinus stiffness, raising the threshold and reducing their responsiveness to pressure changes. Sinoaortic denervation can cause temporary or prolonged hypertension [27,28].

### 2.2. Antidiuretic Hormone

Antidiuretic hormone (ADH), also known as vasopressin, is synthesized in the hypothalamus, primarily by neurons in the supraoptic nuclei and, to a lesser extent, by neurons in the paraventricular nuclei, which mainly produce oxytocin. ADH is transported along the hypothalamic–hypophysial tract to the posterior pituitary, where it is released into the bloodstream via fenestrated capillaries. ADH production and release are triggered by several factors: high plasma osmolarity (detected by osmoreceptors in the hypothalamus), low blood volume (causing reduced tension in low-pressure baroreceptors), decreased blood pressure (leading to reduced stretch in high-pressure baroreceptors), and angiotensin II.

**Figure 1:**
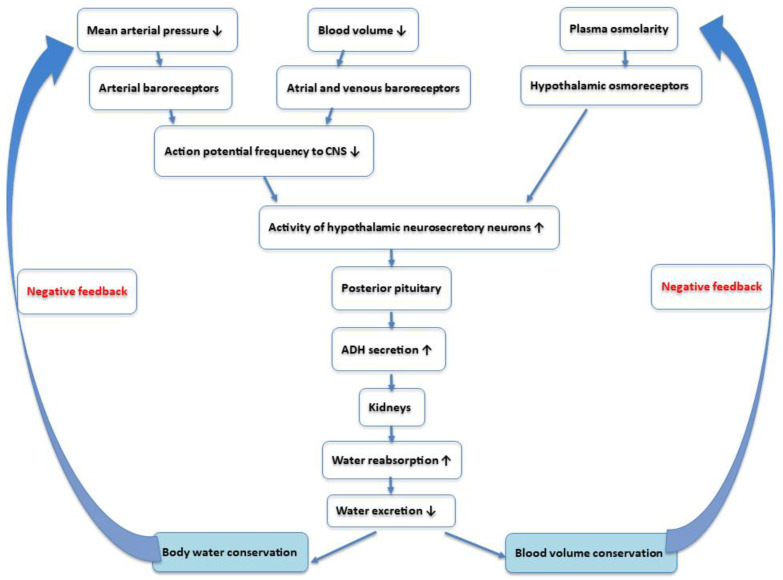
Mechanisms controlling the secretion of antidiuretic hormone (ADH, or vasopressin) and its effects on the body’s water volume, and consequently, on the circulating volume, blood pressure, and body fluid osmolarity. The black arrows indicate the direction of change in physiological parameters.

The primary function of ADH is to increase water reabsorption in the collecting ducts of the kidneys, raising plasma volume and arterial pressure. High concentrations of ADH can also induce moderate vasoconstriction, which increases peripheral resistance and further raises arterial pressure [29,30]. A detailed description of ADH action is reported in Figure 1.

### 2.3. Renin–Angiotensin–Aldosterone System (RAAS)

The renin–angiotensin–aldosterone system (RAAS) plays a critical role in regulating arterial blood pressure by increasing blood volume and peripheral resistance through hormonal actions. It begins when juxtaglomerular cells in the kidneys release renin in response to low blood pressure, increased sympathetic activity, or reduced sodium levels in the distal convoluted tubules. Renin converts angiotensinogen, produced by the liver, into angiotensin I, which is then converted into angiotensin II in the pulmonary vessels by the angiotensin-converting enzyme (ACE).

**Figure 2:**
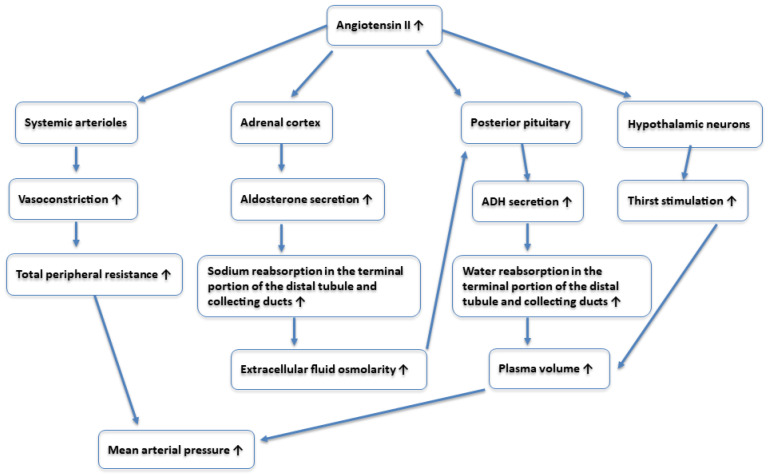
Angiotensin II mechanisms of blood pressure control. The black arrows indicate the direction of change in physiological parameters.

Angiotensin II raises arterial pressure by acting as a potent vasoconstrictor, constricting efferent arterioles in the kidney glomerulus to maintain the glomerular filtration rate, increasing sodium reabsorption in the kidneys (leading to water retention), and stimulating the release of ADH from the posterior pituitary and aldosterone from the adrenal cortex. Aldosterone increases sodium reabsorption and potassium secretion in the kidneys, enhancing water retention and further raising blood pressure. A detailed description of angiotensin II action is reported in Figure 2.

While the baroreceptor reflex provides short-term pressure regulation, the RAAS handles both acute and chronic regulation.

The goal of arterial pressure regulation is to maintain adequate tissue perfusion without causing damage. In cases of acute hypotension, the baroreflex works to stabilize blood pressure. Chronic hypertension, often without a clear cause, is known as essential hypertension and accounts for around 95% of hypertension cases. This condition can lead to severe complications in the brain, heart, and kidneys [31,32,33].

## 3. Search Strategy and Study Selection

The search for studies was conducted using the PubMed database, employing the following search string: “moringa oleifera” [tiab] AND (“blood pressure” [tiab] OR “hypertension” [tiab]). Additional studies were retrieved from the references of the selected studies. The search included studies published up to December 2024. To be included, studies had to be original articles published in English, investigating the effects of *Moringa oleifera* (in its whole form, extract, or specific component) on blood pressure in either animal or human models, or examining the mechanisms through which *Moringa oleifera* might modulate blood pressure.

## 4. Effect of *Moringa oleifera* on Blood Pressure

### 4.1. Animal Studies

Various studies have investigated both acute and chronic effects of *M. oleifera* on blood pressure in both healthy and hypertensive animal models. These experiments, detailed in Table 1, primarily involved the administration of *M. oleifera* leaves, seeds, or their extracts.

Abrogoua et al. [34] tested the hypotensive effect of an aqueous extract, including salt, *Bidens pilosa* whole plant, and fresh *M. oleifera* leaves (20% of weight), on healthy rabbits. The extract, administered via injection, induced a dose-dependent reduction in blood pressure. Similarly, Aekthammarat et al. [35] observed that intravenous administration of the aqueous extract of *M. oleifera* leaves at doses of 1, 3, 10, and 30 mg/kg caused a rapid and dose-dependent reduction in the mean arterial pressure in normotensive rats and in rats pretreated with *N*ω-nitro-L-arginine methyl ester (L-NAME) to induce hypertension. In the normotensive rats, the highest dose of *M. oleifera* extract produced the greatest reduction in blood pressure (−30%), maintained for 37 min. Treatment with the leaf extract also reduced the mean arterial pressure in the hypertensive rats, though to a lesser extent than in the normotensive rats (−8.5% at the 30 mg/kg dose). In addition, Faizi et al. [36] assessed the acute hypotensive activity of ethanolic and aqueous extracts of *M. oleifera* pods, seeds, and leaves, along with their relative fractions and isolates, in normotensive WKY rats. They discovered that the ethanolic extracts of whole pods and seeds, administered at a dose of 30 mg/kg, led to reductions of 41% and 43%, respectively, in the mean arterial blood pressure. Specifically, the neutral and acidic subfractions of the ethyl acetate phase of the ethanolic extract of *M. oleifera* pods exhibited antihypertensive effects, while the aqueous fraction was inactive. β-sitosterol, methyl ester of p-hydroxybenzoic acid, and p-hydroxybenzaldehyde were identified among the isolates from these extracts as having a hypotensive effect. Additionally, extracts in the hot water of pods and leaves also demonstrated hypotensive activity. Regarding the chronic effect, Attakpa et al. [37] investigated the impact of the aqueous extract of *M. oleifera* leaves on male normotensive and spontaneous hypertensive rats (SHRs). The animals underwent a pretreatment with a standard diet for 16 weeks, and then were divided into groups receiving increasing amounts of *M. oleifera* leaves extract (200, 400, and 600 mg/kg) for 8 weeks. They noted a dose-dependent reduction in blood pressure in the SHRs, with significant hypotensive effects observed at doses of 400 and 600 mg/kg compared to the control diet, without a significant difference between the two doses. No effect of *M. oleifera* was observed in the WKY rats. Aekthammarat et al. [38] administered the aqueous extract of *M. oleifera* leaves to normotensive and hypertensive-induced WKY rats at doses of 30 or 60 mg/kg/day for three weeks. Hypertension was induced by adding 50 mg/kg/day of L-NAME to the drinking water for the same duration. They observed a dose-dependent decrease in systolic and diastolic blood pressure, as well as heart rate, in the hypertensive rats treated with *M. oleifera* compared to the untreated rats. However, treatment with *M. oleifera* did not affect blood pressure or heart rate in the normotensive rats. Acuram et al. [39] conducted a study on the antihypertensive activity of methanolic and ethyl acetate extracts of *M. oleifera* leaves in female mice. The animals were divided into several groups and treated for 45 days. Group 1 received distilled water orally (control group); group 2 received solvent or vehicle; group 3 was administered captopril (20 mg/kg/day); groups 4 and 5 received low and high doses of methanol extract of *M. oleifera* (0.01 g/kg/day and 0.3 g/kg/day); groups 6 and 7 were given low and high doses of ethyl acetate extract of *M. oleifera* (0.01 g/kg/day and 0.3 g/kg/day). Hypertension was induced in groups 2–7 by L-NAME treatment (30 mg/kg/day). The L-NAME treatment significantly increased blood pressure. The methanolic extract of *M. oleifera* led to a progressive and significant reduction in blood pressure, comparable to that of the control group by the end of the treatment period. Similar results were observed with the ethyl acetate extracts of *M. oleifera*, although a significant effect was only observed at the end of the treatment period. There was no significant difference in blood pressure between the high (0.3 g/kg/day) and low (0.01 g/kg/day) doses of *M. oleifera* extracts. Chen et al. [40] investigated the acute and chronic effects of *M. oleifera* leaf extract in the WKY rats with induced pulmonary hypertension via subcutaneous injection of monocrotaline (MCT, 60 mg/kg). Acute administration of 4.5 mg/kg of the *M. oleifera* extract reduced pulmonary arterial pressure to around 80% of the MCT control. Chronic supplementation with the *M. oleifera* extract for one week at the same dose reversed the MCT-induced increase in pulmonary arterial pressure. Finally, Randriamboavonjy et al. [41] studied the impact of *M. oleifera* seed powder on cardiac function in SHRs. The animals were divided into two groups: one receiving a standard diet and the other a standard diet supplemented with 750 mg/d/rat of *M. oleifera* seed powder for 8 weeks. The treatment did not alter the diurnal or nocturnal systolic and diastolic arterial pressures. However, it led to a reduction in the nocturnal heart rate, without affecting the diurnal rate, when the animals were awake and active.

Numerous studies have validated the antihypertensive properties of *M. oleifera*, especially its leaves. However, evidence supporting the effects of other parts, like the seeds and pods, is limited. Moreover, the antihypertensive effects appear more pronounced in hypertensive animal models, while results in healthy animals are more varied.

### 4.2. Human Studies

We only found four studies examining the effects of *M. oleifera* on blood pressure in humans. The first research was conducted on healthy individuals, while the other three studies focused on participants with diabetes. Notably, none of the studies included subjects with hypertension. A detailed summary of human study is reported in Table 2.

Chan Sun et al. [42] reported that the consumption of cooked *M. oleifera* leaves led to a reduction in postprandial blood pressure among healthy subjects. This study was a prospective, placebo-controlled clinical trial involving a random sample of 41 healthy participants. Those in the treated group consumed 120 g of the plant’s cooked leaves, while the control group received a placebo. Blood pressure measurements were taken at baseline (prior to the meal) and at regular intervals over the next 24 h (2, 4, 6, 10, 20, and 24 h after the meal). Additionally, all participants maintained a food diary during the week preceding the study, allowing researchers to estimate their total daily salt intake. The results show a significant decrease in diastolic blood pressure from baseline to the 2 h postprandial evaluation in the experimental group, but not in the control group. There were no significant differences observed in systolic blood pressure between baseline and postprandial measurements in either group. Moreover, among the participants in the experimental group with a high salt intake (7 g/day) during the week prior to the study, there was a notable decrease in both their systolic and diastolic blood pressure following the consumption of *Moringa oleifera* leaves. Conversely, higher blood pressure values were recorded in the participants with increased salt consumption in the control group. Afiaenyi et al. [43] employed a double-blind, parallel-group randomized controlled design for their study. The research involved 40 diabetic subjects, comprising 12 men and 28 women, who were randomized into four groups. The control group received an isocaloric diet based on the caloric distribution recommended by the American Diabetes Association (50–60% of daily caloric intake from carbohydrates, 25–30% from fat, and 15–20% from protein) [44]. The experimental groups received daily doses of 20 g, 40 g, and 60 g of Moringa leaves, respectively, in addition to the prescribed diet, for a duration of 14 days. The study findings revealed a notable reduction in systolic blood pressure (by −13 mm Hg, from 136.40 ± 7.66 to 123.90 ± 13.82 mm Hg) in the group treated with 40 g of *M. oleifera* leaves. However, this reduction was not observed in the other groups. Taweerutchana et al. [45] conducted a prospective randomized placebo-controlled study involving individuals with diabetes. The participants were randomly allocated to receive either 8 g per day of *M. oleifera* leaf capsules or a placebo for a duration of 4 weeks. At the conclusion of the treatment period, the researchers did not observe significant changes in systolic or diastolic blood pressure in either the treatment group or the placebo group. Finally, Diaz-Prieto et al. [46] conducted a double-blind, randomized, placebo-controlled, parallel-group study involving 73 patients with prediabetes. The 12-week intervention consisted of a daily administration of six capsules, each containing 400 mg of *M. oleifera* dry leaf powder or a placebo. At the end of the study, no significant effects were observed on systolic or diastolic blood pressure. 

**Table 2:** Effects of *Moringa oleifera* on blood pressure in human studies.

Human Studies			
Studies	Part of Moringa	Experimental Model	Results
Chan Sun et al. [42]	Cooked leaves	A total of 41 participants aged 18–65 years (14 males, 27 females) were randomized into two groups: The experimental group (*n* = 23) consumed 120 g of sautéed *M. oleifera*; The control group (*n* = 18) consumed an equivalent amount of placebo. SBP and DBP were measured at baseline and postprandially at intervals up to 24 h (2, 4, 6, 10, 20, and 24 h after the meal).	Both the case and control groups showed decreased systolic blood pressure postprandially, but no significant difference was observed compared to baseline. Diastolic blood pressure decreased overall postprandially for both groups, with a significant difference noted between 2 h and baseline only in the experimental group.
Afiaenyi et al. [43]	Steamed leaves	A total of 40 adults (12 males, 28 females) with type 2 diabetes were split into groups and treated for 14 days: Group 1: Standard diet; Group 2: Standard diet + 20 g/day *M. oleifera* leaves; Group 3: Standard diet + 40 g/day *M. oleifera* leaves; Group 4: Standard diet + 60 g/day *M. oleifera* leaves.	Group 3 showed a significant decrease in SBP from 136.40 ± 7.66 mm Hg to 123.90 ± 13.82 mm Hg, with a mean decrease of 12.5 mm Hg. Group 4 did not show a significant reduction in SBP (−7.8 mm Hg). No significant reduction in DBP was observed in group 3 or 4 (−4.7 mm Hg and 3.3 mm Hg, respectively).
Taweerutchana et al. [45]	Leaf powder	RCT with placebo involving 32 people with type 2 diabetes. They were treated for 4 weeks with 8 g/day of *M. oleifera* leaf powder (experimental group) or placebo (control group).	No effect on SBP and DBP was observed.
Diaz-Prieto et al. [46]	Leaf powder	A double-blind RCT with placebo involving 73 patients with prediabetes. Patients were treated for 12 weeks with six capsules, each containing 400 mg of *M. oleifera* dry leaf powder (experimental group) or placebo (control group).	No effect on SBP and DBP was observed.

## 5. Possible Mechanisms of Action

Several studies have proposed different physiological mechanisms to explain the hypotensive effects of *M. oleifera* leaves and seeds.

**Figure 3:**
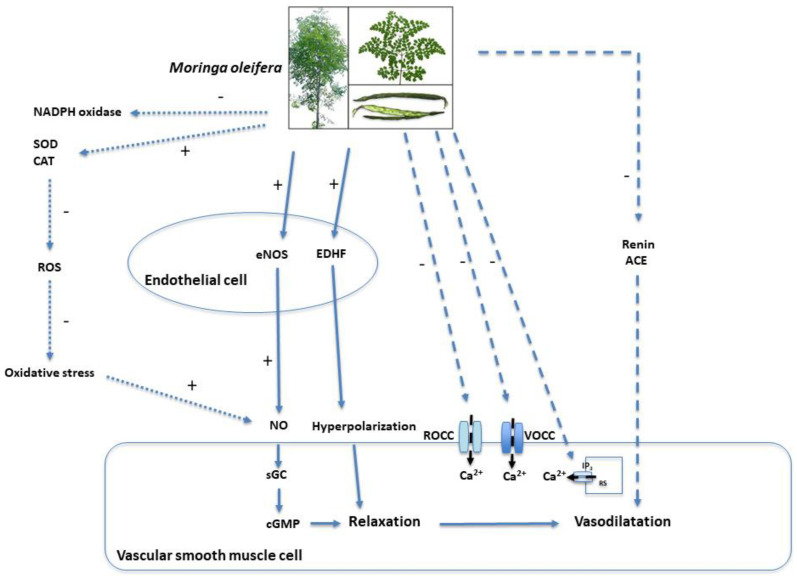
Overview of the mechanisms by which *Moringa oleifera* influences blood pressure. Abbreviation: eNOS, endothelial nitric oxide synthase; NO, nitric oxide; EDHF, endothelium-derived hyperpolarizing factors; sGC, soluble guanylate cyclase; cGMP, cyclic guanosine monophosphate; VOCCs, voltage-operated calcium channels; ROCCs, receptor-operated calcium channels; IP3, inositol trisphosphate, ACE, angiotensin-converting enzyme; SOD, superoxide dismutase; CAT, catalase; ROS, reactive oxygen species.

It has been suggested that *M. oleifera* can induce vasorelaxation through both endothelium-dependent and endothelium-independent mechanisms. These mechanisms are summarized and shown in Table 3 and Figure 3, respectively.

Endothelium-dependent relaxation is a fundamental process in the regulation of vasodilation, mediated by the release of three major factors: nitric oxide (NO), prostacyclin (PGI_2_), and endothelium-derived hyperpolarizing factors (EDHFs) [47]. These mediators act on vascular smooth muscle cells (VSMCs), promoting relaxation and facilitating blood flow. Several experimental studies have indicated that *M. oleifera* leaf extract induces vasodilation in isolated mesenteric arterial beds from both healthy and hypertensive rats [35,38]. The vasodilatory effect of the *M. oleifera* extract is largely endothelium-dependent, as demonstrated by a significant but not complete reduction in efficacy following endothelial removal [35,38]. This suggests a predominant, though not exclusive, role of endothelial mediators in its hypotensive action. The vasodilatory effect of *M. oleifera* leaf extract appears to be linked to its ability to stimulate NO production [35] and reduce oxidative stress [38]. In particular, a study on human pulmonary artery endothelial cells treated with increasing concentrations of *M. oleifera* leaf extract have shown an enhancement in NO production, an effect abolished by L-NAME, a non-selective inhibitor of endothelial nitric oxide synthase (eNOS) [35]. This finding suggests that *M. oleifera* leaf extract activates eNOS, increasing NO availability. A further study on aortic sections from middle-aged rats indicated that the eNOS activation induced by *M. oleifera* seed extract might be mediated by the upregulation of protein kinase B (Akt), which phosphorylates and activates eNOS, and the downregulation of arginase-1. Reduced arginase-1 expression increases the availability of L-arginine, the substrate required for NO biosynthesis, thereby contributing to the vasodilatory effect of the *M. oleifera* extracts [48].

Oxidative stress impairs endothelial function by reducing NO bioavailability due to its reaction with superoxide anion (O_2_^−^), leading to the formation of peroxynitrite (ONOO^−^), a potent oxidant that uncouples eNOS and inhibits its Akt-mediated activation [49,50]. A study demonstrated that treatment with *M. oleifera* leaf extract reduced O_2_^−^ production and enhanced the activity of antioxidant enzymes, such as superoxide dismutase (SOD) and catalase (CAT), which neutralize free radicals [38]. Increased SOD activity was also confirmed in hypertensive rats treated with *M. oleifera* seed extract, which additionally reduced the expression of regulatory subunits of NADPH oxidase, an enzyme involved in ROS production [51]. These findings suggest that *M. oleifera* extract may reduce oxidative stress, thereby increasing NO availability. This, in turn, promotes the relaxation of VSMCs by activating soluble guanylate cyclase (sGC), leading to the production of cyclic guanosine monophosphate (cGMP), a key mediator of vasodilation [35].

Beyond NO-induced vasorelaxation, *M. oleifera* leaf extract also appears to induce vasodilation via EDHFs [52]. EDHFs act by opening potassium (K^+^) channels in VSMCs, causing K^+^ efflux and inducing membrane hyperpolarization. This hyperpolarization leads to the closure of voltage-operated calcium channels (VOCCs), reducing Ca^2+^ entry and consequently inhibiting VSMC contraction. Experimental evidence has shown that *M. oleifera* leaf extract induces dose-dependent relaxation even in arterial preparations pretreated with L-NAME and indomethacin, inhibitors of eNOS and cyclooxygenases (enzymes responsible for PGI2 synthesis), respectively. However, endothelium-dependent vasodilation was significantly reduced when contraction was induced using a high K^+^ solution, a condition that inhibits EDHF-mediated responses [52]. This suggests that *M. oleifera* leaf extract induces vasodilation through a mechanism involving VSMC membrane hyperpolarization. The exact identity of EDHFs remains unclear, but a study suggested that hydrogen sulfide (H_2_S) may be involved [53].

Nonendothelium-dependent mechanisms are physiological processes that regulate vascular function without directly involving the endothelium, but act directly on VSMCs. They primarily involve calcium homeostasis, ion channel activity, and neurogenic control of vascular function. The contractile state of VSMCs is governed by intracellular calcium (Ca^2+^) levels, which are regulated by VOCCs, receptor-operated calcium channels (ROCCs), and intracellular calcium stores controlled by inositol trisphosphate (IP3) receptors. An increase in intracellular Ca^2+^ triggers vasoconstriction, whereas its reduction leads to relaxation. Another critical component of non-endothelium-dependent regulation is neurogenic control, particularly the adrenergic system, where sympathetic activation enhances vasoconstriction via α₁-adrenoceptor activation, whereas NO-containing nerves counteract this effect through axo-axonal inhibition of adrenergic signaling. *M. oleifera* leaf extract has been shown to exert direct effects on VSMCs via non-endothelium-dependent mechanisms [52]. Recent studies on hypertensive rat models have demonstrated that *M. oleifera* leaf extract reduces vascular tone by inhibiting extracellular Ca^2+^ influx and intracellular Ca^2+^ mobilization, leading to decreased contractility. Specifically, *M. oleifera* leaf extract blocks VOCCs and ROCCs, thereby limiting the Ca^2+^ entry required for contraction. Additionally, *M. oleifera* leaf extract modulates IP3-mediated calcium release from the sarcoplasmic reticulum, further attenuating intracellular Ca^2+^ accumulation. Notably, while some vasodilatory agents act by opening K^+^ channels, the most recent findings indicate that MOE-mediated relaxation does not involve potassium channel activation, as evidenced by the fact that K^+^ channel blockers did not alter its vasodilatory effect. Instead, *M. oleifera* leaf extract primarily functions as a calcium channel blocker, displaying a mechanism comparable to that of nifedipine, a well-characterized VOCC inhibitor [52]. Furthermore, in L-NAME-induced hypertension, *M. oleifera* leaf extract has been found to mitigate neurogenic vasoconstriction, likely through oxidative stress reduction, which may preserve NO availability from perivascular nerves and counteract sympathetic overactivation.

Another proposed mechanism to explain the blood pressure-lowering effects of *M. oleifera* is the inhibition of the renin–angiotensin–aldosterone system. Several studies have tested the ACE-inhibitory effect of different protein fractions and phenolic compounds [39,54]. One study [54] found that protein hydrolysates from *M. oleifera* leaves, obtained through enzymatic digestion with Alcalase, exhibit significant inhibitory activity against both angiotensin-converting enzyme (ACE) and renin, with maximum inhibition rates of 84.71% and 43.72%, respectively, for peptides with a molecular weight of <1 kDa. Two bioactive peptides, Leu-Gly-Phe-Phe (LGF) and Gly-Leu-Phe-Phe (GLFF), were isolated and characterized, showing IC_50_ values of 0.29 mM and 0.31 mM for ACE, along with concurrent renin inhibition. Their oral administration to spontaneously hypertensive rats led to a reduction in both systolic and diastolic blood pressure, with the most pronounced effect observed 6 h post-administration, suggesting their potential as natural antihypertensive agents. Another study [39] focused on the methanolic and ethyl acetate extracts of *M. oleifera* leaves, assessing their ability to inhibit ACE in vitro. The ethyl acetate extract demonstrated the highest inhibitory activity and was, therefore, subjected to biofractionation to identify the compound(s) responsible for its bioactivity. This process led to the isolation of two flavonoids, quercetin-3-O-glucoside and kaempferol-3-O-glucoside, which were identified using HPLC and NMR spectroscopy. Notably, quercetin-3-O-glucoside exhibited dose-dependent ACE inhibition, reaching 75.74% at 28 µg/mL. However, the bioactivity of kaempferol-3-O-glucoside could not be assessed due to its low yield.

These findings highlight that the hypotensive effects of *M. oleifera* appear to result from a combination of endothelium-dependent and independent mechanisms, primarily involving NO- and EDHF- mediated vasorelaxation, oxidative stress reduction, calcium channel blockade, and renin–angiotensin system inhibition. Experimental studies highlight its ability to enhance endothelial function, reduce vascular resistance, and modulate neurogenic and hormonal pathways involved in blood pressure regulation.

## 6. Compounds with Hypotensive Potential

The hypotensive properties of *Moringa oleifera* have been studied since the 1990s, with the isolation of various bioactive compounds from leaves and pods using ethanolic and aqueous extracts. The first study [55] isolated, through ethanolic extraction of fresh leaves, thiocarbamate glycosides, including niazinin A, niazinin B, niazimicin, and the mixture of niaziminin A and B. These compounds, purified via TLC and HPLC, exhibited dose-dependent hypotensive activity when injected into normotensive Wistar rats. At a dose of 1 mg/kg, all compounds reduced blood pressure by 14–22%, whereas at 3 mg/kg, the reduction ranged from 40% to 65%. A second study [56] expanded research on ethanolic leaf extracts, isolating an isothiocyanate, such as 4-[(4’-O-acetyl-α-L-rhamnosyloxy)benzyl]isothiocyanate, thiocarbamate glycosides, including niaziminin A, and niaziminin B, as well as two nitrile glycosides, niazirin and niazirinin. In vivo tests in normotensive Wistar rats confirmed that only (4-[(4’-O-acetyl-α-L-rhamnosyloxy)benzyl]isothiocyanate, niaziminin A, and niaziminin B) were active, reducing blood pressure by 15–20% at 1 mg/kg and by 35–40% at 3 mg/kg, whereas the nitriles (niazirin and niazirinin) showed no hypotensive effects up to 5 mg/kg. A third study [57] isolated, from ethanolic leaf extracts, two carbamate glycosides, including niazimin A, and niazimin B, and two thiocarbamate glycosides, including niazicin A and niazicin B. Following intravenous injection into normotensive Wistar rats, niazimin A, niazimin B, niazicin A, and niazicin B reduced blood pressure by 15–20% at a dose of 1 mg/kg, while at 3 mg/kg, the reduction was 35–40%. A fourth study [58] confirmed the role of thiocarbamate glycosides. O-methyl, 4-[(2′,3′,4′-tri-O-acetyl-α-L-rhamnosyloxy)benzyl]thiocarbamate, O-ethyl, 4-[(2′,3′,4′-tri-O-acetyl-α-L-rhamnosyloxy)benzyl]thiocarbamate, and niazimicin B were isolated from the ethanolic extract of *Moringa oleifera* leaves. After administration via the jugular vein in normotensive Wistar rats, all thiocarbamates reduced blood pressure by 10–20% at a dose of 1 mg/kg, while at 3 mg/kg, the reduction ranged from 30% to 40%. In parallel, research extended to the plant’s pods and seeds. A further study [36] examined the hypotensive activity of ethanolic and aqueous pod extracts. The aqueous extract exhibited hypotensive effects at 30 mg/kg. Fractionation of the ethanolic extract led to the isolation of several hypotensive compounds. p-Hydroxybenzaldehyde showed the highest efficacy, reducing blood pressure by 40% at 3 mg/kg and by 75% at 10 mg/kg. Methyl p-hydroxybenzoate, at a dose of 10 mg/kg, caused a 47% reduction, while β-sitosterol, at 3 mg/kg, led to a 43% decrease. Tests further revealed that hypotensive activity was greater in seeds (43% at 30 mg/kg) than in the pericarp, which exhibited a biphasic response, whereas the pulp was inactive. A further study [59] confirmed significant hypotensive effects of niazinin A, niazinin B, niazimicin, and niaziminin A and B obtained from ethanolic extract of *M. oleifera* leaf. Intravenous administration of these compounds (1–10 mg/kg) in anesthetized rats induced a dose-dependent reduction in both systolic and diastolic blood pressure. To investigate the underlying mechanism, guinea pig atria were pretreated with atropine (1 mg/kg), which abolished the hypotensive response to acetylcholine but did not affect the response to the Moringa compounds, ruling out muscarinic receptor involvement. Additionally, a further in vitro experiment on rabbit aorta showed reductions in the contraction force and rate, while in isolated rabbit aorta preparations, the compounds inhibited K^+^-induced contractions. These findings suggest that the hypotensive effect of these compounds is primarily due to a direct depressant action on cardiac and smooth muscle tissues.

More recent studies have investigated the hypotensive properties of the protein fraction [54] and certain phenolic compounds [39]. As previously described, specific peptides (LGF and GLFF) and a flavonoid glycoside (quercetin-3-O-glucoside) have shown ACE-inhibitory effects.

Finally, it is widely known that *M. oleifera* is a plant rich in bioactive compounds, including β-carotene, phenolic acids, flavonoids, and isothiocyanates, which confer significant antioxidant properties [6]. Several studies have demonstrated that *Moringa oleifera* leaf extract effectively reduces oxidative stress by neutralizing excessive ROS, which are responsible for cellular damage and chronic inflammation. This antioxidant capacity is particularly relevant for cardiovascular health, as oxidative stress plays a key role in the development of hypertension and other cardiovascular diseases.

These studies demonstrate that *M. oleifera* contains numerous hypotensive compounds, with thiocarbamates and isothiocyanates as the primary contributors to the effect. Additionally, certain phenols and sterols contribute to the hypotensive activity, although efficacy varies depending on the plant part and the type of extract used.

## 7. Limitations of Using *Moringa oleifera* as an Antihypertensive Therapy

*M. oleifera* possesses a potential antihypertensive effect. However, the use of *M. oleifera* as an alternative therapeutic for hypertension must consider several limitations. Several preclinical studies have suggested the hypotensive potential of *M. oleifera*. However, translating results from animal studies to humans is challenging. Differences in metabolism, cardiovascular regulation, and drug absorption among species can significantly impact how *M. oleifera* affects blood pressure in humans compared to animal models. Additionally, the doses used in animal studies may not be feasible or safe for human consumption. Most animal models of hypertension rely on induced conditions that may not fully represent the complexity of human hypertension, making it difficult to predict real-world clinical effects. Therefore, there is a need to demonstrate the hypotensive effects of *M. oleifera* in humans with appropriate clinical studies. Clinical evidence is currently quite limited, and the results of existing studies are conflicting and inconclusive. The studies conducted so far have primarily involved healthy individuals or patients with diabetes, rather than those with diagnosed hypertension. Some trials have indicated a mild reduction in blood pressure, particularly in subjects with high salt intake, while others have reported no significant effects. Given these inconsistencies, the current data do not allow for definitive conclusions about *M. oleifera* as an antihypertensive agent. Further well-designed, randomized controlled trials involving hypertensive individuals are necessary to clarify its efficacy and determine appropriate dosages. Furthermore, to ensure the safe and effective use of *Moringa oleifera*, it is essential to elucidate the mechanism(s) underlying its antihypertensive effects. Only a limited number of studies, primarily conducted by the same research groups, have addressed this topic. Therefore, the current knowledge requires further validation, preferably through independent studies, to confirm and expand upon the existing findings. Moreover, as described above, the pharmacological effect seems to be related to the numerous secondary metabolites present in the leaves, as well as in other parts of the plant. It is important to note that the concentrations of these molecules depend on numerous factors, such as the variety of the plant and the environmental growth conditions [60], making standardization of the product difficult. Therefore, unlike a drug with a standardized concentration, *M. oleifera* may exhibit variable concentrations of these bioactive components, potentially impacting its efficacy. Moreover, there is currently no consensus on the optimal dosage of Moringa required to achieve antihypertensive effects. As a food product, it is likely that a high intake of Moringa is necessary to achieve effects comparable to those of a pharmaceutical drug. However, high consumption may be impractical because Moringa can alter the sensory characteristics of foods [13], particularly their taste, which could limit individuals’ willingness to consume it in sufficient quantities for a therapeutic effect. Additionally, high intake may cause gastrointestinal side effects [11], limiting its feasibility as a long-term therapy. This can be obviated by identifying the specific molecule(s) responsible for the hypotensive effect. While some studies—mostly outdated—have investigated this aspect, several important considerations must be taken into account. The effects of these molecules have primarily been evaluated using in vitro assays (e.g., enzyme or cell-based assays) or through intravenous administration, bypassing the issue of bioavailability. However, it is well established that secondary metabolites, such as polyphenols and isothiocyanates, exhibit low bioavailability when administered orally. Therefore, in addition to validating previous findings, future research should focus on evaluating the hypotensive potential of these compounds following oral administration to better reflect physiological conditions.

All these aspects currently limit the use of *M. oleifera* as a potential therapy for hypertension. Despite these challenges, smaller amounts of *M. oleifera* could be useful for hypertension prevention or as an adjunct to antihypertensive therapy. However, for this latter point, it is crucial to ensure that there are no interactions with medications, particularly antihypertensive drugs, and to confirm the absence of adverse effects on liver or kidney function. Studies in this area are almost entirely lacking, and therefore, caution is advised when considering *M. oleifera* as an antihypertensive therapy.

## 8. Conclusions and Future Directions

Preclinical studies support the hypotensive properties of *M. oleifera*. Animal studies suggest that various parts of *M. oleifera*, particularly its leaves, may contribute to blood pressure reduction through multiple mechanisms, including NO and EDHF modulation, calcium channel inhibition, and regulation of the renin–angiotensin–aldosterone system. These effects appear to be associated with the presence of thiocarbamates, isothiocyanates, flavonoids, peptides, and other secondary metabolites. However, findings from human studies are limited and inconsistent. Notably, there is a lack of randomized controlled trials specifically targeting hypertensive patients or assessing blood pressure as a primary outcome, which would be necessary to confirm preclinical results.

Further investigations are needed to elucidate the exact mechanisms of action and to identify the specific bioactive compounds responsible for the antihypertensive effects of *M. oleifera*. Given that human administration occurs orally, it is crucial to consider the bioavailability of these compounds, as this could significantly influence their efficacy. Moreover, determining the optimal dosage at which *M. oleifera* exerts its antihypertensive effects is essential to ensure both efficacy and safety. Additionally, for safe clinical use, potential interactions between *M. oleifera* and antihypertensive medications must be thoroughly examined. Addressing these critical aspects will be fundamental to defining the therapeutic potential of *M. oleifera* in hypertension management.

In conclusion, *Moringa oleifera* shows potential as a natural antihypertensive agent, but current evidence is insufficient to support its widespread use for this purpose. Further rigorous research is essential to validate its efficacy, ensure its safety, and establish standardized guidelines for its application in clinical settings.

## Figures and Tables

**Table 1:** Effects of *Moringa oleifera* on blood pressure in animal studies.

Studies	Part of Moringa	Experimental Model	Results
Abrogoua et al. [34]	Aqueous extract of *Bidens pilosa* and fresh leaves of *M. oleifera*	Healthy rabbits were treated with increasing doses of the traditional dietary supplement (5 × 10^−8^ to 5 × 10^−2^ mg/kg bw) or acetylcholine.	The extract produced a dose-dependent hypotensive effect, reducing blood pressure by 7.14 ± 4% to 100 ± 7.5% compared to normal levels in rabbits.
Aekthammarat et al. [35]	Aqueous leaf extract	Six male Wistar rats received acetylcholine (control), followed by leaf extract (1, 3, 10, 30 mg/kg). Six male Wistar rats were pretreated with L-NAME, followed by the same leaf extract doses.	Acute intravenous injection of *M. oleifera* extract caused a dose-dependent reduction in the mean arterial pressure in the normotensive rats, with reductions of 12.87 ± 1.45%, 21.52 ± 1.27%, 27.83 ± 0.64%, and 29.87 ± 2.34%, respectively. Significant differences were observed at all doses compared to 1 mg/kg. L-NAME pretreatment significantly reduced the BP-lowering effect of the extract, with reductions of 2.31 ± 0.61%, 6.09 ± 0.78%, 8.43 ± 1.23%, and 8.57 ± 1.43% at the respective doses.
Faizi et al. [36]	Ethanolic and hot water extracts and relative fractions and isolates of pods, seeds, pulp, and leaves	Normotensive Wistar rats were treated with fractions (10 and 30 mg/kd) and isolates (3 and 10 mg/kg) of *M. oleifera* pod, seed, pulp and leaf extract.	Ethanolic extracts of *M. oleifera* pods and seeds reduced blood pressure by 41% and 43% at 30 mg/kg. The aqueous fraction was inactive. The neutral and acidic sub-fractions of the ethyl acetate phase reduced pressure by 37.6% and 25.5%. Isolated compounds also lowered blood pressure. Hot water extracts of pods and leaves showed similar effects at 30 mg/kg.
Attakpa et al. [37]	Aqueous leaf extract	A total of 20 Wistar–Kyoto normotensive rats (WKYs) and 20 spontaneously hypertensive rats (SHRs) followed a control diet for 16 weeks, then were divided into four groups and treated for 8 weeks. Group 1 was treated only with diet. Groups 2 to 4 received a control diet + *M. oleifera* leaf extract (200, 400, and 600 mg/kg).	The extract reduced BP in the SHR rats in a dose-dependent manner. Significant differences were found between group 2 and group 3, but not between group 3 and group 4. No effects were seen in the normotensive rats.
Aekthammarat et al. [38]	Aqueous leaf extract	A total of 48 male Wistar rats were split into 6 groups. Groups 1 and 2 were normal rats, while groups 3 to 6 were L-NAME-induced hypertensive rats. They were treated daily for 3 weeks with the following: Group 1: distilled water (1 mL/kg/day); Group 2: *M. oleifera* extract (60 mg/kg); Group 3: distilled water (1 mL/kg/day); Group 4: Captopril (5 mg/kg); Group 5: *M. oleifera* extract (30 mg/kg); Group 6: *M. oleifera* extract (60 mg/kg).	Daily intragastrically administration of *M. oleifera* leaf extract decreased the SBP in a dose-dependent manner in the L-NAME hypertensiverats. The highest dose resulted in a lowering of pressure as early as one week into treatment. *M. oleifera* extract (60 mg/kg/day) did not affect blood pressure or heart rate in the normal rats.
Acuram et al. [39]	Methanolic and ethyl acetate leaf extracts	A total of 35 female ICR mice were divided into 7 groups and treated for 25 days: Group 1: Control—distilled water; Group 2: Solvent/vehicle; Group 3: Captopril (20 mg/kg/day); Group 4: Methanol extract (0.3 g/kg/day); Group 5: Methanol extract (0.01 g/kg/day); Group 6: Ethyl acetate extract (0.3 g/kg/day); Group 7: Ethyl acetate extract (0.01 g/kg/day). Hypertension was induced in groups 2–7 by L-NAME treatment (30 mg/kg/day).	Methanolic extract reduced SBP from 102.35 ± 1.55 mm Hg to 90.97 ± 0.80 mm Hg. The ethanolic extract significantly reduced SBP to values comparable to those in the control group. No significant difference in BP was observed at either dosage (0.01 g/kg/day and 0.3 g/kg/day)
Chen et al. [40]	Leaf extract	**Acute study**: A total of 48 male Wistar rats (26 control and 22 with monocrotaline-induced pulmonary hypertension) were treated for 3 weeks with 1.5, 4.5, or 15 mg/kg of *M. oleifera* extract. **Chronic study**: A total of 22 male Wistar rats (7 control and 15 with monocrotaline-induced pulmonary hypertension) were treated for 3 weeks. On days 14–20, the control group received saline injection, and half of the monocrotaline-induced hypertensive rats received 4.5 mg/kg of *M. oleifera* extract.	**Acute study**: In the control rats, no significant difference was found in pulmonary arterial pressure at any dose. In the monocrotaline-induced hypertensive group, pulmonary arterial pressure decreased after Moringa extract administration. The 4.5 mg/kg dose resulted in an 80% reduction in pulmonary arterial pressure. **Chronic study**: Compared to the control group, MCT administration increased pulmonary arterial pressure. Repeated administrations of Moringa extract during the last week significantly reversed the monocrotaline-induced hypertension to a level similar to that of the control group.
Randriamboavonjy et al. [41]	Seed powder	A total of 12 spontaneous hypertensive rats were treated for 8 weeks. The experimental group received *M. oleifera* seed powder mixed into food (750 mg/d/rat), while the control group received normal food.	The treatment had no effect on diurnal and nocturnal SBP and DBP in the hypertensive rats. The treatment reduced the nocturnal heart rate during wakefulness.

**Table 3:** Summary of the mechanisms of action by which *Moringa oleifera* influences blood pressure.

Category	Mechanism		Effect	Final Result
Endothelium-Dependent	(+) eNOS		(+) NO biosynthesis	(+) Vasorelaxation
	(+) Protein kinase B (AKT)			
	(-) Arginase-1			
	(-) Reactive oxygen species (ROS)	(-) oxidative stress	(+) NO availability	
	(+) Superoxide dismutase (SOD)			
	(+) Catalase (CAT)			
	(-) NADPH oxidase			
	(+) Endothelium-derived hyperpolarizing factors (EDHF) bioavailability		Hyperpolarization of VSMC	
Non-Endothelium-Dependent	(-) Receptor-operated calcium channels (ROCCs)		(-) Intracellular Ca^2+^ in VSMC	
	(-) Voltage-operated calcium channels (VOCCs)			
	(-) Inositol trisphosphate (IP3) receptor			
Systemic Regulatory Mechanisms	Inhibition of the renin–angiotensin–aldosterone system (RAAS)		(-) Systemic vascular resistance	
